# "Open Air" and Tuberculosis

**Published:** 1900-02-10

**Authors:** 


### The Hospital

A Journal of

The Medical Sciences and Hospital Administration.

Vol. XXVII.—No. 698.] Edited by Sir Henry Burdett, K.C.B., and Solomon C. Smith, M.D., M.R.C.P. [February 10, 1900.

### "Open Air" and Tuberculosis.

A century ago, when the whole science of domestic hygiene was thought to be comprised in the avoidance of "draughts" and the maintenance of stuffiness, the *Edinburgh Review* first appeared upon the literary horizon, to be followed, a few years later, by the *Quarterly*. The admirably condensed information on a variety of subjects which was afforded by the rival publications was hailed by many enthusiasts as something which would extinguish sciolism, and be incompatible with the continued existence and propagation of error. Others, more sceptical, were of opinion that the aforesaid condensed information would flow through the heads of readers like water through a tin pipe, "leaving them as empty as it found them." If neither of these views has been completely justified by the event, each is still capable of being supported by plausible argumentation; and the second of them should at least place us upon our guard against expecting too much, in the way of popular instruction, from any of the forms in which it may be possible to present truth under a clothing of typography. It does not follow, however, that any such presentation need be wholly thrown away, and we have seen few recent publications more calculated to be instructive to that large section of the public which takes an intelligent interest in medical questions than the report, just issued in a separate form by the Royal Medical and Chirurgical Society, of the discussion on the open-air treatment of tuberculosis, which occupied three meetings of the society in November and December last.

"In a multitude of counsellors," said Solomon, "there is safety;" and it may fairly be assumed that the twenty-three eminent gentlemen who took part in the discussion, and who had evidently prepared themselves to do so with a care commensurate to its importance, left no considerable aspect of the question unexamined. The general result may not unfairly be summed up by saying that there is no "open air" treatment in the popular sense of the words. Tuberculosis depends on the invasion of the body by a bacillus, which flourishes chiefly in badly lighted and badly aërated localities, and languishes, comparatively speaking, when exposed to fresh air and sunshine. These agencies, although detrimental to the invaders, are eminently advantageous to the invaded, and their proper application will suffice, in a large proportion of cases seen at a sufficiently early stage, to turn a nearly balanced scale in favour of the forces of resistance. In other words, the bacilli will die out instead of the patient, and, after the lapse of a period, measurable more often by years than by months, the effects of the invasion will be so far recovered from as to permit a resumption of the occupations, and, to some extent, even of the freedoms, of health. It is obvious that the conditions thus laid down are beyond the reach of all but the comparatively wealthy, and that the necessity which would generally compel members of the industrial classes to resume remunerative occupations as soon as their strength would permit must in every case present a formidable impediment to the real and permanent usefulness of special "sanatoria" maintained by the charitable as hospitals for the tuberculous poor. It is less obvious, but is rendered none the less clear by the discussion, that the rich will not be likely to derive benefit from resort to so-called "sanatoria" in which the medical supervision and control are insufficient, or in which inmates are allowed to please themselves with regard to matters on which it is necessary, if good is to be done, that they should submit implicitly to the directions of the physician. The temperature to which they should be exposed, the kind and amount of food which they should consume, the extent to which they should mix socially with others, and the degree in which they should be kept at rest or permitted to be active, are all questions which require individual determination for each patient; and in respect of which the laxity of a "dividend-earning" establishment would frequently be fatal to every prospect of improvement. These, unfortunately, are among the very points as to which silly and obstinate inmates would persist, we will not say in judging for themselves, but in acting on their own inclinations without the preliminary of a judgment which it would be beyond their power to form. The discussion further afforded interesting evidence, in the speeches of Mr. Pearce Gould and of the President, how much earlier the value of fresh air in tuberculous affections was recognised by surgeons than by physicians; and reminded us of the way in which the late Mr. Solly, when St. Thomas's Hospital was in occupation of temporary premises in which the separation of the medical from the surgical wards was incomplete, used to complain of the difficulty of securing proper ventilation for his cases. "The physicians," he said, "think foul air is a specific for phthisis."

